# Pandemic Lessons for Future Nursing Shortage: A Prospective Cohort Study of Nurses' Work Engagement before and during 16 Months of COVID-19

**DOI:** 10.1155/2023/6576550

**Published:** 2023-07-06

**Authors:** Monique M. A. Penturij-Kloks, Simon T. de Gans, Mandy van Liempt, Esther de Vries, Fedde Scheele, Carolina J. P. W. Keijsers

**Affiliations:** ^1^Jeroen Bosch Academy, Jeroen Bosch Hospital, P.O. Box 90153, 5200 ME's-Hertogenbosch, Netherlands; ^2^Strategy Department, Jeroen Bosch Hospital, P.O. Box 90153, 5200 ME's-Hertogenbosch, Netherlands; ^3^Jeroen Bosch Academy Research, Jeroen Bosch Hospital, P.O. Box 90153, 5200 ME's-Hertogenbosch, Netherlands; ^4^Tranzo, TSB, Tilburg University, P.O. Box 90153, 5000 LE Tilburg, Netherlands; ^5^School of Medical Sciences, Amsterdam UMC, Location VUmc, P.O. Box 7057, 1007 MB, Amsterdam, Netherlands; ^6^Department of Geriatric Medicine, Jeroen Bosch Hospital, P.O. Box 90153, 5200 ME's-Hertogenbosch, Netherlands

## Abstract

**Aim:**

To measure how nurses' work engagement developed during the first three COVID-19 waves and to compare this with the data collected just before the outbreak.

**Background:**

The shortage of nurses is a threat to population health. COVID-19 posed nurses with personal and professional challenges that affected their work engagement. Insights into how the pandemic affected their work engagement may help hospitals retain and recruit nurses in the future.

**Methods:**

A single centre prospective survey study was conducted using the UWES-9.

**Results:**

In total, 1,697 nurses (90.5% female, mean age 41 years) completed four assessments. Each assessment showed a significant decrease in work engagement compared with that before COVID-19. Work engagement stabilized in the last two assessments.

**Conclusion:**

Work engagement decreased significantly compared with that in March 2020, just before the outbreak. Although the decrease stabilized from the 8^th^ month to the 16^th^ month, it did not return to pre-COVID-19 levels. Whether this stabilization was the beginning of a recovery in work engagement or reflected a permanent decline needs to be established. *Implications for nursing management*. Nurse leaders should facilitate nurses' self-regulation processes and encourage them to develop resources in order to maintain work engagement at a high level.

## 1. Background

Nurses have an important role in population health and wellbeing [1]. They interact directly with patients, and their performance has a great impact on the quality of care and patient outcomes [2], with more nursing staff being associated with better patient outcomes [3]. However, there is a global nursing shortage [4]. The nursing workforce is ageing and experiencing high levels of work-related stress, which has led to early retirement and nurses leaving the profession to pursue a different career [4].

During the COVID-19 pandemic, nurses faced an unprecedented number of professional challenges such as caring for critically ill patients, fearing becoming infected and infecting relatives, coping with a limited availability of personal protective equipment, and changing nursing and care protocols, which led to a massive increase in the demands of work [5, 6]. Working under stressful conditions for long periods of time with little time for recovery poses a serious risk to one's physical and psychological wellbeing [7, 8]. Long-term strategies to safeguard the wellbeing of nurses under similar conditions are essential to prevent deterioration in the quality of care provided, exacerbation of nurses' intention to leave the profession, and deterioration of nurses' work engagement, which is essential because work engagement protects against emotional fatigue and burnout [6, 9, 10]. Engaged employees perform better, as they are more likely to experience positive emotions, develop their resources, have better health, and transfer their engagement to others [11].

Work engagement, a term introduced by Bakker and Schaufeli, is described as a positive, fulfilling, and work-related state of mind that is characterized by three subcategories, namely, vigour, dedication, and absorption [12]. The overall concept of work engagement refers to a persistent and pervasive affective-cognitive state that is not focused on any particular object, event, individual, or behaviour [13]. The subcategory vigour is characterized by high levels of energy and mental resilience while working, the willingness to invest effort in one's work, and persistence even in the face of difficulties. Dedication refers to being strongly involved in one's work and experiencing a sense of significance, enthusiasm, inspiration, pride, and challenge. Absorption is characterized by being fully concentrated and happily engrossed in one's work, whereby time passes quickly and one has difficulty detaching oneself from work [12].

Nurses' work engagement has been widely studied to gain a better understanding of how to improve their performance, patient care and outcomes, and nurse retention [14–16]. Research on work engagement in professional nursing practice often uses the job demands-resources model (JD-R) as a theoretical framework [2, 17]. The JD-R theory uses two categories of job characteristics to characterize a work context, namely, job demands and job resources [18]. Job demands are those aspects of the job that require physical and/or psychological effort (e.g., cognitive demands, work pressure, and others) and are associated with physiological and/or psychological costs [19]. In essence, job demands consume energy because they must be addressed [20]. High or unfavourably designed job demands impair health, resulting in exhaustion and health problems (e.g., anxiety, depression, and post-traumatic stress disorder) [21]. Job resources are functional aspects of the job that are important for achieving work goals, and they reduce job demands and stimulate personal growth, learning, and development (e.g., opportunities for growth and social support) [22]. In essence, job resources are motivational and increase employee work engagement and performance [20].

Did nurses' work engagement change during the COVID-19 pandemic? Many studies have investigated work engagement in relation to psychological distress or risk [5, 6, 9, 23], often based on a single measurement. However, multiple measurements are needed to obtain an understanding of how work engagement changes during a health crisis. There are six longitudinal trajectories in response to stressful events, which are as follows: resistance, resilience, recovery, relapsing/remitting, delayed dysfunction, and chronic dysfunction [24]. An insight into these changes may present opportunities for interventions to increase the work engagement of nurses. To the best of our knowledge, there have been no studies of how nurses' work engagement changed during the first three waves of the COVID-19 pandemic relative to that before the pandemic started.

### 1.1. Objective

The objective of this research was to measure the work engagement of nurses during the first three COVID-19 waves, from just before the first COVID-19 patient was admitted (March 2020) to the last assessment, 16 months later (July 2021). Given the need for multiple measurements to understand the change in nurses' work engagement in health crises, our research questions were further specified as follows: (1) how do the work engagement scores of nurses differ just before and at three measurement points during the following 16 months of the COVID-19 pandemic? (2) What are the scores of the subdomains vigor, dedication, and absorption at these measurement points?

## 2. Methods

### 2.1. Study Design

**Figure 1:**
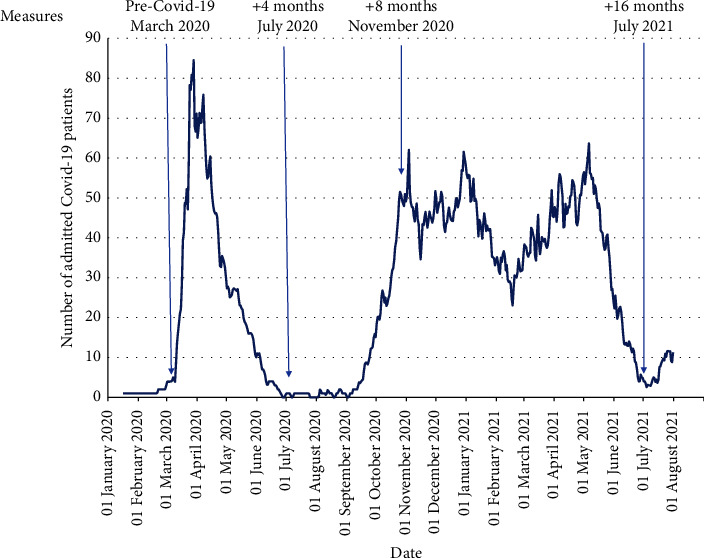
Study design with work engagement assessed before the pandemic (pre-COVID: March 2020) and during the subsequent 16 months (July and November 2020 and July 2021), in relation to the daily number of COVID-19 patients admitted to the Jeroen Bosch Hospital.

This single-centre prospective cohort study measured the work engagement of nurses before and during three COVID-19 waves, a total of 16 months.  Figure 1 shows the study design with assessments in March, July, and November 2020 and July 2021. The first assessment was just before the first COVID-19 wave broke, when only one patient had been admitted.

The timing of the measurement points could not be predicted precisely due to the great uncertainty of the pandemic. To give an example, nobody predicted such a long second wave. Usually the yearly employee measure is just before the summer holiday (July). In 2020, due to worries about health professionals' wellbeing, additional measures were taken.

### 2.2. Variables

The primary study outcome variable was the total score of nurses' work engagement. Secondary outcomes were the subcategories of work engagement, namely, vigour, dedication, and absorption. Predictor variables were age and sex and the number of hospitalized COVID-19 patients (patients per day) over time.

### 2.3. Setting and Study Population

The study was conducted at the Jeroen Bosch Hospital (JBH), a teaching hospital in the Netherlands. All nurses registered at the human resources department were eligible to participate; there were no exclusion criteria. The level of education was not directly measured. However, at all measurement points, only registered nurses worked in the JBH. Therefore, all included participants were registered nurses. At those measurement points, data from the human resources department were used to identify the level of education. Over the measurement points, this was stable, namely, one-third of all nurses (32.3–33.6% over 4 measurement points) have had a higher professional education, while two-thirds have had a secondary vocational education.

Nurses were approached by email and asked to participate. All nurses were asked to complete questionnaires at all assessment times to prevent selection bias. An exception was the first assessment in March 2020, when a random sample of clinical nurses was selected by a blind employee of the human resources department, ten nurses per department. This was done because at that time, multiple surveys were being carried out among nurses.

### 2.4. Data Collection and Tools

All data were collected by an anonymous survey using the online tool (enalyzer.com) [25]. Nurses were sent a link to the online survey by email and, if necessary, they were sent a reminder after 10–14 days of the first email. The data were converted to SPSS version 25 for analysis. Baseline survey characteristics were collected with an open question on age and a dichotomic question on sex. Work engagement was measured with the Utrecht Work Engagement Scale 9 (UWES-9) [12]. It consists of nine questions, covering vigour, dedication, and absorption, scored on a 7-item Likert scale, ranging from 0 “never” to 6 “always.” Work engagement may be considered a one-dimensional (total score of work engagement) as well as a three-dimensional construct (its subscales are vigour, dedication, and absorption). The internal consistency of the three scales of the UWES is good as in all cases, values of Cronbach's *α* are equal to or exceed the critical value of 0.60 [12]. The mean scores were calculated for work engagement overall and for the subcategories in Table 1.

The exposure variable was the daily number of hospitalized COVID-19 patients (over time), as retrieved from the hospital's data management system (Figure 1). The number of admitted COVID-19 patients per day influenced the organization of COVID-19 wards and reallocation of nurses towards these COVID-19 wards.

### 2.5. Statistical Analysis

Respondents with missing UWES-9 data were excluded. Participants had to complete at least one subcategory of UWES-9 answers in order to be included in the analysis. The baseline characteristics were compared with a one-way ANOVA for age and a chi-square test for sex. Any significant differences in the baseline characteristics were added to the main analysis as covariates of adjustment for potential confounders.

The primary outcome work engagement (dependent variable) was analysed on the basis of the total mean and subcategory scores. Different measurement points (independent variables) were compared by linear regression, first a crude analysis, then in an adjusted analysis. Those variates that significantly differed in their characteristics were added as covariates to correct for potential confounding effects. The following comparisons were made: the score before the pandemic was compared with the scores on the other three measure points. Because the only comparisons of our interest were comparisons with the first measurement, data on other between group comparisons, e.g., coefficients of the regression analyses, were not given; just the intercept (constant) was given as a mean (CI). *P* values were shown between crude and adjusted analyses to correct for possible confounding effects; otherwise, does any difference found in intercept still exist after correction for possible confounders?

The dataset was checked for missing data (<30%) before all analyses. For analyses, SPSS version 25 was used. Significance was set at 0.05.

### 2.6. Ethical Approval

The Medical Ethics Committee Brabant gave permission for this study under number NW2020-83. They declared that no human intervention was involved.

## 3. Results

### 3.1. Participants' Characteristics

As shown in Table 2, a total of 1,697 surveys were returned, of which 1,499 could be used for analyses after checking for completeness. The mean age of the participants was 41.2 years and 90.5% were female. Differences in age and sex over the time points were significant and thus added as covariates of the main analyses.

### 3.2. Exposure

We used the number of hospitalized COVID-19 patients per day as a proxy for exposure (Figure 1). The first wave (end of March 2020) was high and steep, with a maximum of 76 COVID-19 patients, and lasted 11 weeks. The second and third waves were lower and longer, with a maximum of 62 COVID-19 patients, and lasted 38 weeks.

### 3.3. Main Results on Overall Work Engagement

Work engagement among nurses decreased significantly at all times compared with March 2020 (Table 3). This decrease remained significant at all times after adjustment for potential confounders, namely, age and sex.

### 3.4. Subcategory Analyses

**Figure 2:**
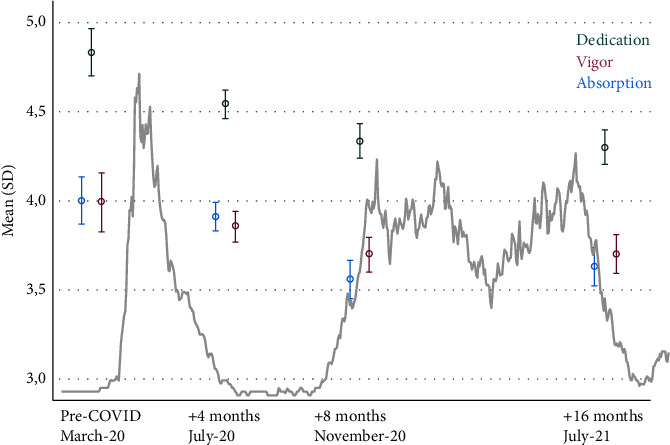
Subcategories (dedication, vigor, and absorption) scores of nurses' work engagement: pre-COVID-19, +4 months, +8 months, and +16 months.

Figure 2 and Table 3 show data for the work engagement subcategories vigour, dedication, and absorption. The subcategory vigour first remained stable (March 2020 mean 4.0 vs. July-2020 mean 3.9, *p* = 0.257), but it declined thereafter in comparison to before the pandemic (March 2020). A similar pattern was observed for the subcategory absorption: in the first months, it remained stable (March 2020 mean 4.0 vs. July 2020 3.9, p 0.118) but decreased later. In contrast, scores for the subcategory dedication decreased immediately.

## 4. Discussion

We found that nurses' work engagement decreased steadily during the 16 months of the COVID-19 pandemic compared with that measured just before the outbreak in March 2020. Table 2 and Figure 2 show that vigour and absorption remained stable in the second measurement, whereas dedication had already declined significantly. In the third and fourth measurements, all subcategories declined significantly compared to the first measurement before the pandemic.

### 4.1. Assessment of the Work Engagement Scores before COVID-19

The UWES manual [12] identifies five qualifications for scoring categories, which are as follows: very high, high, average, low, and very low. The overall work engagement score in this research is classified as “average.” The average score can be found between the 25^th^ and 75^th^ percentiles, and its range is 2.89–4.66 in the UWES norm table. As can be seen, both crude and adjusted scores are in the upper part of the range (4.28 crude and 4.57 adjusted). Many other studies on the work engagement of nurses before COVID-19 have reported comparable average scores (3.3–4.0) [15, 26–29].

### 4.2. Decrease in Work Engagement during the Pandemic

Our results show that work engagement declined significantly and persistently during the COVID-19 pandemic relative to that before the outbreak in March 2020 when nurses were confronted with a wave of seriously ill patients. This is of importance as work engagement is correlated with the three dimensions of burnout—exhaustion, cynicism, and professional inefficacy [30]. Dedication is strongly negatively correlated with cynicism. The correlation between vigour and exhaustion is relatively low, and absorption is the least correlated with the burnout scales [12]. The significant decline in dedication after four months should therefore be highlighted as it is the first item to drop and is highly correlated with burnout. In the next measurement of November 2020, vigour declines significantly as well, adding to the risk of burnout.

Work engagement is regarded as a motivational outcome of an individual's ability to regulate demands and resources [20]. Using the job demands-resources model as a theoretical framework [21], we identified a number of specific job demands during the epidemic, namely, wearing personal protective equipment (PPE), rapidly changing protocols, more patient admissions, caring for critically ill patients, increasing bed capacity, and entering unfamiliar settings [31, 32]. The most important personal and organizational factors associated with nurses' work engagement are workload, mental health, and practice environment [33, 34]. During the pandemic, job demands were associated with psychological challenges such as worry, confusion, nervous mood, and restlessness [32, 35] although COVID-19 pandemic training was found to act as a buffer [36, 37]. Another study found that caring for COVID-19 patients and the situation at work (job demands), transformational leadership, and preparedness (job resources) as well as indicators of strain (chronic fatigue and work satisfaction) were significant determinants of nursing staff turnover [10]. These findings show the impact of specific job demands and resources at a single measurement point in the pandemic but give insufficient insight into the effects of the long duration of the pandemic on work engagement. In a recent article, Demerouti and Bakker, using the job demands-resources model, concluded that in times of crisis, the demands and resources come from various life domains, namely, personal, family, work, and organizational [20]. These demands are interconnected, and individuals continuously have to keep them in balance. The authors formulated an extended demand-resource model based on all life domains which centralized self-regulation processes [20].

We found that, in the early phase of the epidemic, nurses were confronted with high demands and an interplay of job, personal, organizational, and home demands, demands that they found hard to cope with during their long-term exposure to a high number of COVID-19 patients. With an ageing workforce and a high intention among nurses to leave the profession [4], the future will impose more stress on nurses, necessitating organizational support.

### 4.3. Stabilization of Work Engagement after 8 Months

The work engagement score had stabilized between November 2020 and July 2021. This could suggest that nurses' self-regulatory processes had become more effective. Another explanation for this stabilization might be found in the event systems theory [38]. The strength of an event is reflected in its novelty, disruption, and criticality. Before the first wave, the virus was unknown and thus novel [39]. The exponential increase in cases disrupted health care and society [40], and it was a critical event because of its transmission mechanism and the need to act directly [1]. After 8 months, the strength of the event (the pandemic) may have diminished somewhat because the virus was no longer novel and nurses were better prepared after the first two waves [10] and they had a better understanding of how to treat patients and to cope with work disruptions. This could have led to stabilization of work engagement.

A third explanation involves longitudinal trajectories of responses to stress. There are different hypothetical courses of a stress response to bring a system back to the predisaster state, which are as follows: resistance, resilience, recovery, relapsing/remitting, delayed dysfunction, and chronic dysfunction [24]. Resilience and recovery are probably relevant with regard to the stabilization of work engagement. Resilience is defined variously as the process of, capacity for, or outcome of successful adaptation after trauma or severe stress [41]. Recovery characteristically involves a period of dysfunction lasting several months or more, followed by a gradual return to pre-event functioning [42]. The recovery pattern is a gradual resilience pattern as it takes more time [24]. The pattern we see in our results is a significant decline on all measurements compared to before COVID-19 with a stabilization of this decline after 8 months. This stabilization may indicate some recovery, so it might be the case that we are looking at a recovery pattern.

Resilience is regarded as a personal resource in the job demands-resources model [43], and by becoming resilient, employees can become more engaged as they may have greater ability to control their work environment [44]. It is thus important to help nurses become resilient by balancing their individual mix of demands and resources.

When extrapolating these three COVID-19 waves, with an excessive number of patients combined with a nursing shortage, to the future of the nursing profession, we can perhaps conclude that the pandemic was just a general rehearsal. Given that the work engagement of nurses is an important determinant of patient outcomes and experiences, the pandemic highlighted the importance of helping nurses balance the demands of a crisis situation and understand which job resources contribute to their work engagement. Nurse leaders and management should individualize policies to support nurses in this situation.

## 5. Future Research

Whether the work engagement of nurses will bounce back to levels before COVID-19 needs to be established. Given the importance of the self-regulation of job demands and job resources, further research is needed to better understand this process and how it can be facilitated.

## 6. Limitations

This is the first longitudinal study of nurses' work engagement before and during three waves of the COVID-19 pandemic. However, it had some limitations. For legal reasons, it was not possible to ask for employee numbers, so we could not perform a paired analysis. It was a single-centre study, which raised questions about the generalizability of the findings. However, the Jeroen Bosch Hospital had an average UWES score before COVID-19, as had been found in other studies and hospitals, which suggest that the findings are applicable to other hospitals. Lastly, not all nurses were included in the first assessment, before the pandemic broke, which could have led to selection bias even though nurses were selected at random.

## 7. Conclusion

The work engagement of nurses significantly decreased compared to that in March 2020, just before the outbreak. Although the decline stabilized in the period between November 2020 and July 2021, work engagement did not return to prepandemic levels and it was in a recovery trajectory. Whether elements of the disruption of care associated with the pandemic are common to future scenarios needs to be investigated. If so, lessons from this challenge can be used to prepare for future growth in the number of patients and a decline in the number of available nurses. Resilience, a personal job resource, supports work engagement, and this aptitude needs to be developed in nurses by means of organizational support, training, and development [44].

### 7.1. Implications for Nursing Management

Delivering quality care with engaged nurses under challenging circumstances requires that nurse leaders facilitate nurses' self-regulation processes and stimulate different types of job resources in order to maintain work engagement at a high level.

## Figures and Tables

**Table 1:** Norm scores for the UWES-9 [12].

	Vigour	Dedication	Absorption	Total score
Very low	≤2.00	≤1.33	≤1.17	≤1.77
Low	2.01–3.25	1.34–2.90	1.18–2.33	1.78–2.88
Average	3.26–4.80	2.91–4.70	2.34–4.20	2.89–4.66
High	4.81–5.65	4.71–5.69	4.21–5.33	4.67–5.50
Very high	≥5.66	≥5.70	≥5.34	≥5.51

**Table 2:** Participants' characteristics.

	Pre-COVID March-20	+4 months July-20	+8 months November-20	+16 months July-21	*p* value
Employed nurses that were invited (*n*)	286	1052	1065	1040	
Number of nurses employed	1009	1052	1065	1040	
Total surveys received (*n*)	170	691	454	382	
Response rate (%)	59.4%	65.7%	42.6%	36.7%	
Complete and used in analyses (*n*)	170	601	384	344	
Age, mean (SD)	37.6 (13.1)	41.8 (12.9)	42.1 (13.1)	41.0 (12.6)	0.001^*∗*^
Female (%)	95.9%	91.7%	84.4%	92.4%	<0.001^†^
Contractual hours (h/wk), mean (SD)	29.4 (6.0)	28.7 (6.1)	28.7 (6.3)	28.6 (6.0)	0.604^*∗*^

^*∗*^ANOVA, ^†^chi-square.

**Table 3:** Primary outcome work engagement: before COVID-19 versus during the 16 months of subsequent COVID-19 waves by linear regression.

	Pre-COVID March-20	+4 months July-20		+8 months November-20		+16 months July-21	
	Mean (CI)	Mean (CI)	July-20 vs pre-COVID *p*value	Mean (CI)	Nov-20 vs pre-COVID *p*value	Mean (CI)	July-21 vs pre-COVID *p*value
*Overall work engagement*							
Crude	4.28 (4.15–4.40)	4.10 (3.83–4.38)	0.022^*∗*^	3.86 (3.58–4.15)	<0.001^*∗*^	3.88 (3.59–4.17)	<0.001^∗^
Adjusted	4.57 (4.34–4.80)	4.42 (4.03–4.80)	0.041^†^	4.19 (3.80–4.58)	<0.001^†^	4.19 (3.80–4.58)	<0.001^†^

*Subcategory vigour*							
Crude	4.00 (3.86–4.15)	3.91 (3.60–4.89)	0.257^*∗*^	3.54 (3.21–3.86)	<0.001^*∗*^	3.62 (3.29–3.95)	<0.001^*∗*^
Adjusted	4.08 (3.82–4.35)	3.11 (3.55–3.64)	0.258^†^	3.62 (3.18–4.06)	<0.001^†^	3.70 (3.25–4.14)	<0.001^†^

*Subcategory dedication*							
Crude	4.83 (4.69–4.97)	4.53 (4.23–4.83)	<0.001^*∗*^	4.32 (4.00–4.63)	<0.001^*∗*^	4.30 (3.98–4.61)	<0.001^*∗*^
Adjusted	5.06 (4.80–5.31)	4.78 (4.36–5.19)	<0.001^†^	4.57 (4.14–4.99)	<0.001^†^	4.54 (4.11–4.97)	<0.001^†^

*Subcategory absorption*							
Crude	3.99 (3.84–4.14)	3.85 (3.52–4.18)	0.118^*∗*^	3.69 (3.35–4.02)	0.001^*∗*^	3.70 (3.36–3.14)	0.003^*∗*^
Adjusted	4.55 (4.28–4.82)	4.45 (4.00–4.75)	0.253^†^	4.31 (3.58–4.27)	0.011^†^	4.29 (3.83–4.11)	0.007^†^

^*∗*^Crude: dependent: work engagement; independent: measure points. ^†^Crude analyses with adjustment for age and sex.

## Data Availability

The data that support the findings of this study are available on request from the corresponding author.
